# Editorial: Neutrophils in Cancer

**DOI:** 10.3389/fimmu.2022.862257

**Published:** 2022-02-18

**Authors:** Brahm H. Segal, Zvi Fridlender

**Affiliations:** ^1^ Roswell Park Comprehensive Cancer Center, Jacobs School of Medicine & Biomedical Sciences, University at Buffalo, Buffalo, NY, United States; ^2^ Institute of Pulmonary Medicine, Hadassah Medical Center, The Faculty of Medicine, Hebrew University of Jerusalem, Jerusalem, Israel

**Keywords:** neutrophils (PMNs), cancer, tumor microenvironment, myeloid-derived suppressor cell (MDSC), innate immunity

Neutrophils are short-lived immune cells whose main function is to mediate antimicrobial host defense. To function effectively, neutrophils must sense microbial products and host-derived cytokines and chemokines, traffic to sites of infection and injury, and kill pathogens. Neutrophils contain a broad armamentarium of antimicrobial products, many of which are sequestered in granules and activated by infection and mimics of infection (e.g. bacterial cell wall products). In addition to microbial products, neutrophils can be activated by damage-associated molecular patterns (DAMPs) (1–4), which are endogenous danger signals that are released from damaged or necrotic cells and activate the innate immune system by engaging pattern recognition receptors (PRRs). However, the same pathways that kill pathogens and amplify inflammation can also cause injury to the host. Neutrophil extracellular traps (NETs) are extracellular chromatin and granular constituents that are generated in response to microbial and damage motifs, and target extracellular pathogens, but are also pro-thrombotic and injurious (5). The research of the role of “*Neutrophils in Cancer*”, the theme of this Research Topic, has emerged substantially during the last decade. A major theme of this Research Topic, relates to how antimicrobial pathways in neutrophils are activated by DAMPs and cytokine signals in the tumor microenvironment (TME) in ways that can promote or restrict tumor progression. Tumor-associated neutrophils are reprogrammed by cues in the TME, and, in turn, can cross-signal to tumor cells and reshape the immune landscape of the TME. These signaling interactions driven by neutrophils are potential targets for immunotherapy.

A number of studies show that tumor-infiltrating granulocytic cells are negative prognostic biomarkers. Gentles at al. (6) analyzed expression signatures from ~18,000 human tumors with overall survival outcomes across 39 malignancies, and identified intra-tumoral neutrophil signatures as the most significant adverse cancer-wide prognostic marker. Si et al. (7) noted intra-tumoral hotspots for co-localization of granulocytic and T cells in primary head and neck tumors, where a subset of granulocytes expressing LOX-1 and arginase I correlated with reduced activation of adjacent T cells.

The TME can influence neutrophil biology at multiple levels. Tumor-derived factors (e.g., G-CSF or GM-CSF) can drive abnormal myelopoiesis skewed toward granulocytic differentiation. Disordered myelopoiesis is a central mechanism for expansion of myeloid-derived suppressor cells (MDSC) in cancer. MDSCs are a heterogeneous subset of myeloid cells that can have a granulocytic or monocytic lineage, and are defined by their ability to suppress T cell immunity. The distinction between granulocytic MDSC (PMN-MDSC), which are typically defined by acquisition of suppressor function due to abnormal granulopoiesis (8), and normal neutrophils acquiring suppressor function in circulation or in the TME is important conceptually and therapeutically. One approach to limit PMN-MDSC expansion is based on the premise of tumor causing disordered granulopoiesis resulting in release of intrinsically suppressive granulocytes (8). Therapeutic targeting of neutrophils that acquire suppressor function after release from marrow would rely on impairing their trafficking to the TME [e.g., by CXCR2 inhibition (9)] and/or abrogating their suppressor function acquired in the TME.

One of the major themes of this Research Topic relates to neutrophils being recruited and altered by injurious and inflammatory cues in the TME. The similarity between tumor stroma and non-healing wounds has been recognized for decades (10). Normal wound healing following infection, trauma and other acute injuries is characterized by ordered stages of thrombosis, inflammation, and resolution. Neutrophils are the first responders to infection and injury; following resolution of the insult, inflammation transitions to less injurious chronic inflammatory cells (monocytes and lymphocytes). The TME is a pathologic, non-resolving wound characterized by persistent cellular injury and recruitment of mixed populations of inflammatory cells that comprise the immune landscape. Although neutrophil heterogeneity has been recognized for decades (11), the concept of distinct neutrophil populations at the transcriptome and phenotypic level has been advanced with molecular and cellular profiling tools. Neutrophils subsets in the TME can be differentiated at both the transcriptional (12) and metabolic levels (13), and the TME can induce distinct neutrophil clusters (14).

This neutrophil heterogeneity raises the notion for distinct populations accelerating or reducing tumor growth and metastasis. Neutrophils can bind to circulating tumor cells and enhance hematogenous metastasis through enhancing tumor cell cycle progression (15). Activated neutrophils can kill tumor cells through reactive oxidant generation (16, 17) and antibody-dependent cellular cytotoxicity (ADCC). One exciting therapeutic approach involves enhancing ADCC of neutrophils and macrophages directed against tumor cells through inhibition of the SIRPα-CD47 “don’t eat me” pathway (18, 19). In contrast, tumor-infiltrating neutrophils can drive tumor progression and metastasis through a number of pathways, including stimulation of thrombosis and angiogenesis, stromal remodeling, and impairment of T cell-dependent anti-tumor immunity (20, 21). NETs can facilitate tumor progression in tumor-bearing mice through a number of mechanisms including vascular thrombosis, disabling of T cell immunity, and tumor cell signaling (22–24). Finally, although a substantial body of literature has shown that tumor-associated granulocytes can suppress T cell responses, different inflammatory or tumor-derived cues in the TME can prime neutrophil responses that enhance T-cell immunity (25, 26).

This Research Topic addresses key facets of how the TME influences granulocyte biology and how these distinct granulocyte populations influence tumor progression. Kramer and Abrams provide a cutting edge review of mechanisms driving PMN-MDSC generation and their suppressive effects on antitumor immunity. Furumaya et al. probe the concept of neutrophil plasticity in the TME, resulting in neutrophils exerting either pro-tumor or anti-tumor effects and discuss novel therapeutic approaches that target neutrophils. Hsu et al. provide a mechanistic overview of the specific steps where neutrophils can influence tumor growth and metastasis, including local tumor cell invasion and entry into circulation, survival of tumor cells and hematogenous metastatic seeding, and growth of tumor at metastatic sites. Zhang et al. specifically focused on the role of tumor-infiltrating N1 (anti-tumorigenic) and N2 (tumor-promoting, immunosuppressive) neutrophils in breast cancer. Domnich et al. review the role of salivary neutrophils in the development and progression of oral cancer. This review addresses neutrophils in the healthy oral cavity versus their activity in conditions that predispose to oral cancers (e.g., aging, smoking, and chronic periodontitis) and the effects of neutrophils in established oral cancers. Masucci et al. review the role of NETosis in tumor growth and metastasis, including their direct signaling to tumor cells, and highlight the potential application of NET inhibitors in cancer. The principles reviewed in this Research Topic provide an important mechanistic foundation for how neutrophils regulate the TME and for designing therapeutics to enhance tumor control, and add another important layer to our understanding of the immune TME in general, and specifically on the role of neutrophils in the progression of cancer.

## Author Contributions

Both authors contributed to the article and approved the submitted version.

## Funding

NIH R01CA188900-01 (BS).

## Conflict of Interest

The authors declare that the research was conducted in the absence of any commercial or financial relationships that could be construed as a potential conflict of interest.

## Publisher’s Note

All claims expressed in this article are solely those of the authors and do not necessarily represent those of their affiliated organizations, or those of the publisher, the editors and the reviewers. Any product that may be evaluated in this article, or claim that may be made by its manufacturer, is not guaranteed or endorsed by the publisher.
